# Supplementary material for: Drug repurposing for aging research using model organisms
Source: Aging Cell. 2017 Jun 16;16(5):1006–15. doi: 10.1111/acel.12626 (PMC5595691; doi:10.1111/acel.12626)
Supplement: Supplementary file 7 — Data S1 Zip‐Archive of all report cards. [file ACEL-16-1006-s007.zip › RC_AFE.pdf]

AFE

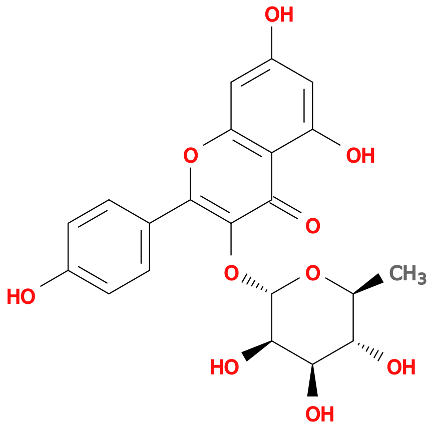

Database identifiers

ChEMBLCompound	CHEMBL240528
CHEBI	80790
ZINC	ZINC15657732
eMolecules	6854804

Ranking

	Rank	Score
Drosophila	202/697	0.697
C. elegans	172/591	0.265

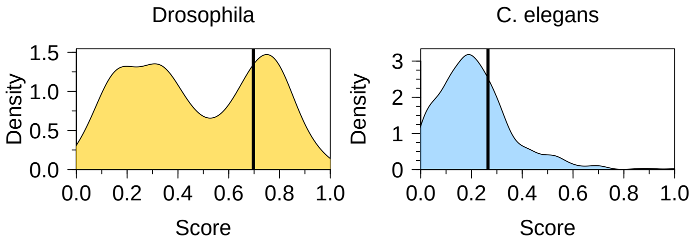

	Ageing implication	Domain conservation	Binding site conservation	Binding affinity	Bioavailability	Lipinski	Promiscuity	Purchasability	Drug approval	**Total**
Drosophila	1.0	0.924	0.984	0.792	(0.9)	-0.05	-0.0	0.1	0.0	0.697
C. elegans	1.0	0.955	1.0	0.792	0.285	-0.05	-0.0	0.1	0.0	0.265

Names

- **afzelin**
- Kaempferin
- Kaempferol 3-O-alpha-L-rhamnoside

Roles

plant metabolite, antibacterial agent

Status

Approved drug (according to ChEMBL)	No
Number of Rule of 5 violations	1
Binding affinity to original target in log units (RF-Score prediction)	6.34
Burns *C. elegans* bioavailability prediction	-0.79

Compound Target Characteristics

Ribosomal protein S6 kinase alpha-3

Best gene implication in ageing for this target family came from gene Q86NP0 annotated in UniProt release 2014_02. Annotation GO 8340 (determination of adult lifespan) was Inferred from Mutant Phenotype

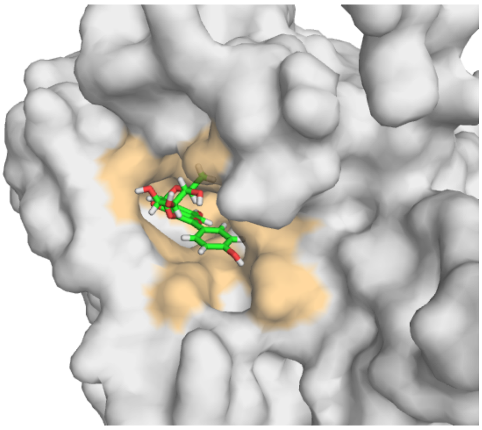

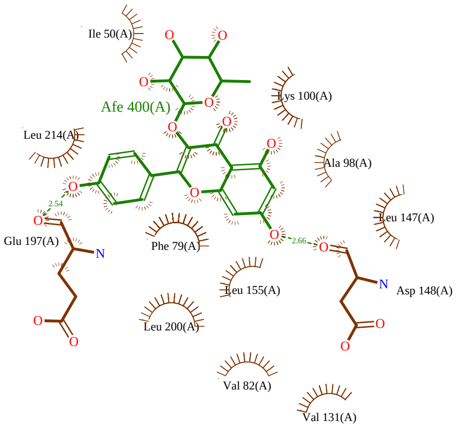

protein	amino acids contacts (binding site)
PDB:4el9:chainA:P18654	I F V A K V L D L E L L
tr:B1AXG1:B1AXG1_HUMAN	I F V A K V L D L E L L
tr:B7Z2K7:B7Z2K7_HUMAN	I F V A K V L D L E L L
tr:B4DG22:B4DG22_HUMAN	I F V A K V L D L E L L
tr:B7ZB17:B7ZB17_HUMAN	I F V A K V L D L E L L
tr:E9PRI4:E9PRI4_HUMAN	I F V A K V L D L E L L
tr:F5GYC4:F5GYC4_HUMAN	I F V A K V L D L E L L
tr:E9PGT3:E9PGT3_HUMAN	I F V A K V L D L E L L
sp:Q15418:KS6A1_HUMAN	I F V A K V L D L E L L
sp:P51812:KS6A3_HUMAN	I F V A K V L D L E L L
tr:F1LXV0:F1LXV0_RAT	I F V A K V L D L E L L
tr:D3Z8E0:D3Z8E0_RAT	I F V A K V L D L E L L
tr:B1AXP0:B1AXP0_MOUSE	I F V A K V L D L E L L
tr:B1AXN9:B1AXN9_MOUSE	I F V A K V L D L E L L
tr:E9PWV3:E9PWV3_MOUSE	I F V A K V L D L E L L
sp:P18653:KS6A1_MOUSE	I F V A K V L D L E L L
tr:F6RQA2:F6RQA2_MOUSE	I F V A K V L D L E L L
tr:Q505N6:Q505N6_MOUSE	I F V A K V L D L E L L
tr:F6Q8A4:F6Q8A4_MOUSE	I F V A K V L D L E L L
sp:P18654:KS6A3_MOUSE	I F V A K V L D L E L L
tr:Q810V8:Q810V8_MOUSE	I F V A K V L D L E L L
tr:Q86NP0:Q86NP0_DROME	F F V A K V L D L E L L
tr:Q9VR61:Q9VR61_DROME	F F V A K V L D L E L L
tr:Q24496:Q24496_DROME	F F V A K V L D L E L L
sp:Q21734:KS6A1_CAEEL	I F V A K V L D L E L L
tr:G5EE78:G5EE78_CAEEL	I F V A K V L D L E L L

protein	whole protein ident	simil	domain-based ident	simil	contact-based ident	simil
PDB:4el9:chainA:P18654	1.0	1.0	1.0	1.0	1.0	1.0
tr:B1AXG1:B1AXG1_HUMAN	0.29	0.3	0.67	0.69	1.0	1.0
tr:B7Z2K7:B7Z2K7_HUMAN	0.66	0.75	0.83	0.91	1.0	1.0
tr:B4DG22:B4DG22_HUMAN	0.96	0.96	0.97	0.97	1.0	1.0
tr:B7ZB17:B7ZB17_HUMAN	0.96	0.96	0.97	0.97	1.0	1.0
tr:E9PRI4:E9PRI4_HUMAN	0.78	0.9	0.83	0.91	1.0	1.0
tr:F5GYC4:F5GYC4_HUMAN	0.94	0.95	0.92	0.94	1.0	1.0
tr:E9PGT3:E9PGT3_HUMAN	0.75	0.86	0.79	0.87	1.0	1.0
sp:Q15418:KS6A1_HUMAN	0.76	0.87	0.83	0.9	1.0	1.0
sp:P51812:KS6A3_HUMAN	1.0	1.0	1.0	1.0	1.0	1.0
tr:F1LXV0:F1LXV0_RAT	0.76	0.87	0.83	0.9	1.0	1.0
tr:D3Z8E0:D3Z8E0_RAT	1.0	1.0	1.0	1.0	1.0	1.0
tr:B1AXP0:B1AXP0_MOUSE	0.27	0.28	0.63	0.64	1.0	1.0
tr:B1AXN9:B1AXN9_MOUSE	0.96	0.96	0.97	0.97	1.0	1.0
tr:E9PWV3:E9PWV3_MOUSE	0.78	0.91	0.83	0.91	1.0	1.0
sp:P18653:KS6A1_MOUSE	0.75	0.86	0.8	0.87	1.0	1.0
tr:F6RQA2:F6RQA2_MOUSE	0.77	0.9	0.82	0.92	1.0	1.0
tr:Q505N6:Q505N6_MOUSE	0.76	0.87	0.83	0.9	1.0	1.0
tr:F6Q8A4:F6Q8A4_MOUSE	0.76	0.87	0.83	0.9	1.0	1.0
sp:P18654:KS6A3_MOUSE	1.0	1.0	1.0	1.0	1.0	1.0
tr:Q810V8:Q810V8_MOUSE	0.73	0.83	0.83	0.9	1.0	1.0
tr:Q86NP0:Q86NP0_DROME	0.45	0.67	0.67	0.85	0.92	0.98
tr:Q9VR61:Q9VR61_DROME	0.46	0.69	0.67	0.85	0.92	0.98
tr:Q24496:Q24496_DROME	0.46	0.69	0.67	0.85	0.92	0.98
sp:Q21734:KS6A1_CAEEL	0.55	0.8	0.74	0.91	1.0	1.0
tr:G5EE78:G5EE78_CAEEL	0.54	0.78	0.74	0.91	1.0	1.0

S6kII (FBgn0262866) associated phenotypes

dominant, learning defective, male limited, memory defective, neuroanatomy defective, reduced, short lived

(Information from FlyBase)

rskn-1 (UniProt:Q21734) annotation

Function: Serine/threonine kinase that may play a role in mediating the growth-factor and stress induced activation of transcription.
Cofactor:Mg(2+)
Enzyme regulation: Activated by multiple phosphorylations on threonine and serine residues.
Ptm: Autophosphorylated on Ser-422, as part of the activation process.

(Information from UniProt)

rskn-1 (UniProt:G5EE78) annotation

Cofactor: Mg(2+) Evidence=(PIRNR:PIRNR000606);

(Information from UniProt)
